# Comparison of two procedures for symptomatic hemorrhoidal disease: Ligation under Vision and Ferguson Hemorrhoidectomy - a retrospective cohort study

**DOI:** 10.12669/pjms.331.11266

**Published:** 2017

**Authors:** Hakan Demir, Kerem Karaman, Metin Ercan, Havva Belma Kocer, Fehmi Celebi

**Affiliations:** 1 Dr. Hakan Demir, Department of General Surgery, Sakarya University Teaching and Research Hospital, Sakarya, Turkey; 2 Kerem Karaman, Associate Professor, Department of General Surgery, Sakarya University Teaching and Research Hospital, Sakarya, Turkey; 3 Metin Ercan, Associate Professor, Department of General Surgery, Sakarya University Teaching and Research Hospital, Sakarya, Turkey; 4 Prof. Havva Belma Kocer, Department of General Surgery, Sakarya University Teaching and Research Hospital, Sakarya, Turkey; 5 Prof. Fehmi Celebi, Department of General Surgery, Sakarya University Teaching and Research Hospital, Sakarya, Turkey

**Keywords:** Hemorrhoid, Transanal hemorrhoidal dearterialization, Doppler transducer, Ligation under vision

## Abstract

**Objective:**

To compare Ligation under Vision (LUV) with Ferguson Hemorrhoidectomy (FH) in patients with Grade II, III and IV hemorrhoidal diseases according to their postoperative outcomes.

**Methods:**

Between July 2008 and August 2014, 155 patients underwent FH and 120 patients LUV, in Sakarya University Teaching and Research Hospital. Our retrospective analysis focuses on postoperative complications, postoperative pain and rate of recurrence. In LUV procedure, submucosal tissue of the hemorrhoidal pile base was transfixed using absorbable sutures under direct vision through anoscope in the Jackknife position.

**Results:**

In a mean postoperative follow-up period of 51.76+/-22.3 months; ectropion, anal fissure, and anal incontinence were the most frequent complications. The overall complication rate was significantly less after LUV than FH, (6.7% *vs*. 14.2%, *P*=0.047). The complication rate and need for a second or third surgery did not significantly differ between the two procedures with the increase in affected quadrants (*P*>0.05). The visual analog scale (VAS) at 24 hours was similar in both groups (*P*=0.267).

**Conclusions:**

LUV is a safe, and practical procedure with similar outcomes compared to FH. LUV may be a better choice than excisional hemorrhoidectomies when three or four quadrants of the anal canal are involved with hemorrhoids as this reduces mucosal defect related possible complications such as ectropion and anal stenosis.

## INTRODUCTION

Since Morinaga et al. first described the Doppler guided Transanal Hemorrhoidal Dearterialization (THD) procedure, it has gained acceptance as a popular non-excisional hemorrhoidectomy operation.^1^ Patients complain of less pain after this procedure, their hospital stay is shorter, and they are able to return to their daily activities much more quickly, This success is likely due to their being no real wound after this procedure in contrast to excisional hemorrhoidectomy techniques.^2^-^4^

In the original THD procedure, a Doppler transducer is used to localize the supplying arteries of the corpus cavernosum recti in the distal rectum. These arteries are ligated through a specially designed proctoscope. However, at a later d0ate, one study described artery ligation without using the Doppler transducer and after comparing results, the authors concluded that the Doppler transducer does not contribute additional benefit.^5^ Further to this, in 2008 Bronstein et al. described the Ligation under Vision (LUV) procedure for the treatment of bleeding hemorrhoids.^6^ This procedure consisted of transfixion of the hemorrhoidal pile base with two or three stitches under direct vision. Since that time, LUV has gained popularity and is performed as one of the regular hemorrhoid operations in our clinic.

The main purpose of the present study was to compare retrospectively the LUV procedure with FH in patients with Grade II, III and IV hemorrhoidal disease, according to postoperative outcome.

## METHODS

Following approval of the Institutional Ethical Board Committee of Sakarya University, patients with Grade II, III and IV hemorrhoidal disease who underwent LUV or FH under elective circumstances were analyzed retrospectively. Exclusion criteria included previous hemorrhoidal surgery; acute thrombosed hemorrhoid; pregnancy; Crohn’s disease; neuromuscular disorders causing anal incontinence; previous rectal surgery due to tumor involvement or presence of concomitant perianal abscess; fistula and colorectal or anal carcinoma.

Our patients’ symptoms were evaluated according to their anamnesis; this comprised pain, bleeding, mucosal prolapse, defecation problems such as constipation, and discomfort in daily life like pruritus and soiling. The grade of the hemorrhoids and the involvement of quadrants of the anal canal were determined by preoperative rectal examination. All this data had been recorded in the patient’s folder.

### LUV procedure

**Fig. 1:**
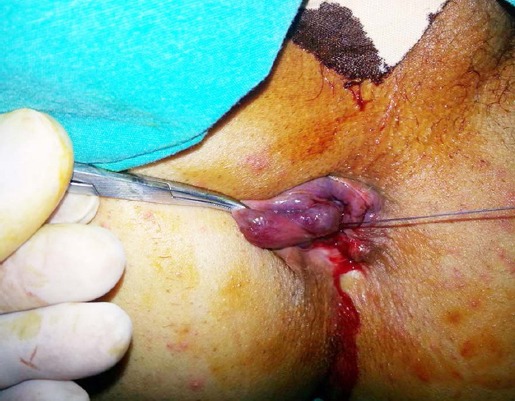
Image of the Ligation under Vision procedure.

The LUV procedure was performed in the operating room under spinal anesthesia using the Jackknife position and exposing the anal canal using the Hill-Ferguson anoscope. Ligations were placed at the base of the area of visible pathologic hemorrhoidal piles just approximately 2 to 3 cm above the dentate line. Ligations were performed with an absorbable braided synthetic 2/0 polyglactin suture (Vicryl, Ethicon Inc, Somerville, NJ, USA). The hemostatic Z suture transfixed mucosal and submucosal layers to include the artery. No additional pexy procedures were performed (Fig.1).

### Postoperative follow-up

The patients were discharged on the first postoperative day and were re-examined in the first postoperative week and at sixth months. Further follow-up was established by rectal examination after telephoning and calling in all the patients who took part in the study. Assessment of postoperative pain was calculated using VAS at 24 hours before discharge. A 0 to 10 numerical rating scale was used to measure the grade of pain in which 0 points equaled “no pain”, and 10 points was the “worst” pain. Points between 0 and 4 were accepted as mild pain; points between 4.1 and 7 as moderate; and points over 7 as severe pain.^7^

### Statistical Analysis

Data analysis was performed by using SPSS for Windows, (version 17.0, Chicago, IL, USA) and the Kolmogorov Smirnov test determined whether the distribution of continuous variables was normal or not. While the continuous variables were shown as mean ± SD or median (min-max), the number and percentage of cases was used for nominal and ordinal data. The mean difference in age between groups was compared using Student’s t test while Mann Whitney U test was used for comparing the median follow-up period and VAS at 24th hour. Nominal data was analyzed by Pearson’s chi-square or Fisher’s exact test, where appropriate. Ordinal variables (i.e. grade, number of affected quadrants and surgical interventions) were also evaluated by Mann Whitney U test. A *P* value of less than 0.05 was considered statistically significant.

## RESULTS

After approval of the ethical committee of the Sakarya University, 275 patients who underwent LUV or FH between July 2008 and August 2014 for Grade II, III and IV symptomatic hemorrhoidal disease at Sakarya University Teaching and Research Hospital, were retrospectively analyzed. Of these, 158 (57.5%) were female and 117 (42.5%) male. The mean age was 40.7+/-13.5 years. Twenty-seven patients (9.8%) were admitted with Grade II hemorrhoids, 139 patients (50.5%) with Grade III, and 109 patients (39.6%) with Grade IV disease. The most common preoperative complaint was bleeding (114 patients: 41.5%); followed by discomfort such as soiling and pruritus (77 patients: 28%); constipation (48 patients: 17.5%); pain (38 patients: 13.8%) and mucosal prolapse (7 patients: 2.5%). Under preoperative rectal examination, hemorrhoidal cushions involving one quadrant of the anal canal were found in 31 patients (11.3%); 2 quadrants in 96 patients (34.9%); three quadrants in 110 patients (40%), and four quadrants in 38 patients (13.8%). 155 patients (56.4%) underwent FH and 120 patients (43.6%) LUV. The mean postoperative follow-up period was 51.76+/-22.3 months. When comparing LUV with FH according to age, gender, preoperative complaints, grade of the hemorrhoidal disease, number of affected quadrants, number of surgical interventions, VAS at 24 hours, postoperative follow-up period and postoperative recurrences, no significant difference was found (*P*>0.05). However, the overall complication rate was significantly less after the LUV procedure than the FH (6.7% *vs*. 14.2%, *P*=0.047) (Table-I).

**Table-I:** Patient’s characteristics.

Variables	LUV (n=120)	FH (n=155)	p-value
Age (years)	40.3±13.6	41.3±13.6	0.566†
Gender			0.083‡
Male	44 (36.7%)	73 (47.1%)	
Female	76 (63.3%)	82 (52.9%)	
Preoperative complaints			
Pain	16 (13.3%)	22 (14.2%)	0.838‡
Bleeding	46 (38.3%)	68 (43.9%)	0.355‡
Mucosal prolapse	3 (2.5%)	4 (2.6%)	1.000§
Defecation Problems¤	22 (18.3%)	26 (16.8%)	0.736‡
Discomfort^#^	34 (28.3%)	43 (27.7%)	0.914‡
Grade			0.228*
II	15 (12.5%)	12 (7.7%)	
III	61 (50.8%)	78 (50.3%)	
IV	44 (36.7%)	65 (41.9%)	
Number of effected quadrants			0.448*
1	13 (10.8%)	18 (11.6%)	
2	44 (36.7%)	52 (33.5%)	
3	51 (42.5%)	59 (38.1%)	
4	12 (10.0%)	26 (16.8%)	
Number of surgical interventions			0.077*
1	108 (90.0%)	128 (82.6%)	
2	11 (9.2%)	23 (14.8%)	
3	1 (0.8%)	4 (2.6%)	
Postoperative complication rate	8 (6.7%)	22 (14.2%)	0.047‡
Recurrence	1 (0.8%)	2 (1.3%)	1.000§
Postoperative follow-up period	60 (6-80)	55 (4-80)	0.387*
VAS at 24 hour	6 (4-7)	6 (4-7)	0.267*

† Student’s t test,

‡ Pearson’s Chi-square test,

§ Fisher’s exact test,

* § Mann Whitney U test,

¤ Constipation,

# Soiling, pruritus.

Postoperative complications developed in 30 patients (10.9%); ectropion and anal fissure being the most common. The distribution of complications according to the groups is listed in Table-II. No anal incontinence, perianal fistula or thrombosis occurred in patients undergoing LUV.

**Table-II:** Postoperative complication types.

Postoperative complications	LUV (n=120)	FH (n=155)
Ectropion	2 (1.7%)	9 (5.8%)
Anal fissure	3 (2.5%)	4 (2.6%)
Anal incontinence	-	2 (1.3%)
Perianal fistula	-	2 (1.3%)
Thrombosis	-	2 (1.3%)
Bleeding	1 (0.8%)	1 (0.6%)
Perianal abscess	1 (0.8%)	1 (0.6%)
More than one complication	1 (0.8%)	1 (0.6%)

The distribution of postoperative complications and the need for second or third surgeries in the two groups did not significantly differ according to the number of involved quadrants (*P*>0.05), (Table-III).

**Table-III:** Distributions of the complications and secondary or third surgery after LUV and FH according to effected quadrants.

	LUV	FH	p-value¶
Complications †	n=8	n=22	0.344
2	1 (12.5%)	2 (9.1%)	
3	6 (75.0%)	12 (54.5%)	
4	1 (12.5%)	8 (36.4%)	
Secondary or third surgery ‡	n=12	n=27	0.776
2	1 (8.3%)	3 (11.1%)	
3	9 (75.0%)	17 (63.0%)	
4	2 (16.7%)	7 (25.9%)	

† Distribution of the complications after LUV and FH according to affected quadrants,

‡ Distribution of the secondary or third surgery after LUV and FH according to effected quadrants,

¶ Mann Whitney U test.

Indications for a second or third surgery were as follows: elective excision of the hemorrhoidal piles extending to the skin due to mucosal prolapse in 13 patients; ectropion in 9 patients; anal fissure in seven patients; bleeding in two patients; thrombosis in two patients; perianal fistula in 2 patients; and perianal abscess in one patient. The necessity for secondary or third surgery was more frequent after FH (Table-IV).

**Table-IV:** Indications for second or third surgery.

Indications	LUV (n=12)	FH (n=27)
Mucosal prolapse	4	9
Ectropion	2	8
Anal fissure	3	4
Bleeding	1	1
Thrombosis	-	2
Perianal fistula	-	2
More than one complication	1	1
Perianal abscess	1	-

## DISCUSSION

Non-excisional hemorrhoidectomy techniques are based on the disruption of the artery flow from the superior rectal arteries which feed the hemorrhoidal plexus in the rectal column. However, these techniques present some challenges. The THD procedure cannot be performed completely, even with Doppler transducer, due to the rich transmuscular collateral network of the rectal vessels.^8^ Moreover, beside the surgeon’s experience, the need for a specialized instrument limits the widespread use of THD. Another non-excisional operation called the Longo technique (stapled hemorrhoidopexy) has been demonstrated as less painful, but less effective in preventing recurrences, and not cost effective. Moreover, rare but serious complications like stenosis, rectal perforation, and recto-vaginal fistulas may occur as a result of this procedure.^9^-^12^ On the other hand, LUV is a simple and cost-effective treatment option for the surgeon, which does not require specialist training and allows for other procedures to be performed later if necessary.^6^,^13^

In the current study, LUV was performed in Grade II, III and IV hemorrhoidal diseases, with Grade III patients composing the largest group. The complication rate after the LUV procedure was 6.7%, which is quite acceptable when compared to similar studies (Table-V).^3^,^5^,^6^,^13^-^23^ None of the chronic complications such as anal incontinence and perianal fistula occurred after the LUV procedure. Furthermore, the need for second and third surgical interventions was less after the LUV procedure than the FH.

**Table-V:** Studies including non-excisional hemorrhoidectomy techniques.

	Study Design	Number of Patients	Comparison (n)	Complication or Failure (%), (P)	Patient Outcomes
Bronstein et al., 2008	Retrospective	32 LUV	-	9%^†^	well
Gupta et al., 2008	Retrospective cohort	616 LUV	-	9%	well
Pakravan et al., 2009	Retrospective	38 TOH	-	10%	well
Schuurman et al., 2012	RCT	82	non-Doppler THD (40) vs THD (42)	0% vs 7.1% (< 0.0005)	Similar
Elmer et al., 2013	RCT	40	THD (20) vs OEH (19)	5% vs 21% (NS)*	Similar
De Nardi et al., 2014	RCT	50	THD (25) vs OEH (25)	12.5% vs 4.3% (NS)^☼^	Similar
Denoya et al., 2014	RCT	27	THD (12) vs OEH (15)	0% vs 13.3% (NS)	Similar
Ratto et al., 2015	Retrospective cohort	803	THD (803)	9.3%^†^	Similar
Tsunoda et al., 2015	Retrospective	66	THD (36) vs OEH (30)	5.6% vs 20% (NS)	Similar
Karaca et al., 2015	Retrospective	47 LUV	-	19.1%	well
Labella et al., 2015	Prospective observational	108 THD	-	8%^$^	well
Present study	Retrospective	275	LUV (120) vs OEH (155)	6.7% vs. 14.2% (<0.047)	Similar

* Complications at 1 year follow-up,

† Failure rate of treatment,

$ Not satisfaction at 1 year follow-up,

☼ Not satisfaction at 2 years follow-up

LUV: Ligation under vision, NS: Statisticallly non-significant difference, OEH: Open excisional hemorrhoidectomy, RCT: Randomised controlled trial, THD: Transanal hemorrhoidal dearterialization, TOH: Transanal open hemorrhoidopexy

None of our patients developed anal stenosis. This is probably related to the avoidance of unnecessary ligation sutures around the entire rectal column, which may disturb rectal arterial flow. Most complications such as anal fissure, thrombosed external hemorrhoids, and prolonged pain may also relate to the deteriorated blood circulation within the mucosa of the anal canal.^3^ Thus, ligation of only the supplying arteries of the hemorrhoidal tissue is more effective than ligating all rectal arteries in the rectal column.^1^ Another factor which may prevent occurrence of anal stenosis after the LUV procedure, is to leave the hemorrhoidal tissue behind after transfixion sutures. This leads to spontaneous shrinkage of the hemorrhoidal piles without the development of a mucosal tissue defect and makes LUV superior to excisional hemorrhoidectomies in this setting.

Results of the present study show that the occurrence of ectropion was far more frequent after FH than after LUV. Moreover, the postoperative complication rate after FH increased with the number of affected quadrants; anal incontinence developed after FH in two patients in whom four quadrants were affected. So, it seems that, LUV should be preferred to FH in patients with three or four affected quadrants, regardless of the grade, to reduce possible complications such as anal stenosis, ectropion or incontinence.

One disadvantage of the LUV procedure is the high probability of the persistence or occurrence of a mucosal prolapse. In the present study, elective excision of hemorrhoidal piles was required in four patients (3.3%) after the LUV procedure due to mucosal prolapse. Although we did not use them ourselves, the addition of mucopexy sutures is recommended by some authors.^16^-^18^,^20^,^24^

It has been shown by some previous studies that postoperative pain is lower after the THD procedure.^3^,^15^,^25^ In contrast to these studies, the VAS scale in the current study was a little higher after the LUV procedure. This may be related to a temporary venous congestion in the hemorrhoidal piles after ligation.

A major advantage of the present study is the large volume of the study population and the long postoperative follow-up period. Secondly, the original nature of this study stems from its comparison of LUV to excisional hemorroidectomy technique, in contrast to previous papers which focused on LUV and its outcomes alone.^6^,^13^,^22^ Additionally, beside the grade of the hemorrhoidal disease, the number of involved quadrants of the anal canal and their role in the development of complications is also evaluated. On the other hand, the study has some limitations. First, it is not blinded and retrospective. Secondly, cost analysis was not performed.

In conclusion, LUV is a safe, and practical procedure. Routine ligation of all arteries in the rectal column may be unnecessary and ligation of only the visible hemorrhoidal cushions seems to be feasible. The outcome is similar both after LUV and FH. LUV may be preferred to excisional hemorrhoidectomies if three or four quadrants of the anal canal are involved with hemorrhoids, to reduce mucosal defect related possible complications such as ectropion and anal stenosis. Clearly, further prospective randomized studies are required to reach a definite conclusion.
